# Rapid Correction of Hyponatremia With Isotonic Saline Leading to Central Pontine Myelinolysis

**DOI:** 10.7759/cureus.38342

**Published:** 2023-04-30

**Authors:** Sulhera Khan, Sonia Das, Wajeeha Batool, Bareerah S Khan, Marium Khan

**Affiliations:** 1 Dermatology, Dow University of Health Sciences, Civil Hospital Karachi, Karachi, PAK; 2 Internal Medicine, Jinnah Postgraduate Medical Centre, Karachi, PAK; 3 Internal Medicine, Dow University of Health Sciences, Civil Hospital Karachi, Karachi, PAK

**Keywords:** locked-in syndrome, treatment of hyponatremia, hyponatremia, integration of palliative care service, central pontine myelinolysis (cpm)

## Abstract

Central pontine myelinolysis (CPM) is a part of the spectrum of osmotic demyelination syndrome (ODS), which is a rare demyelinating disorder due to the rapid correction of low serum sodium. It affects the neurons of the pons but may also involve other extra-pontine sites. The disease is characterized by a wide variety of clinical features ranging from dysarthria, dysphagia, bulbar palsy, quadriplegia, and behavioral and psychiatric disturbances. We present a case of a young female who developed CPM due to rapid sodium correction after vomiting. She presented with quadriplegia and locked-in syndrome. The diagnosis is on the basis of clinical and radiographic features. Magnetic resonance imaging (MRI) of the brain is superior to computed tomography (CT) in detecting changes. It shows hyperintensities on T2-weighted images that are classically known as the trident sign in the region of the pons. The patient was managed supportively, and the family was counseled regarding the poor prognosis of the disease. Unfortunately, she met a fatal fate due to a complication of CPM that is aspiration pneumonia. It is, therefore, imperative to create more awareness regarding the disease, and measures should be taken for its prevention that includes correction of low sodium levels not greater than 10 mmol/L/day.

## Introduction

Central pontine myelinolysis (CPM), a part of osmotic demyelination syndrome (ODS), was first described in 1959 by Adam and Victor [1]. Two forms of ODS are known, one of which is CPM and the other is extra-pontine myelinolysis [2]. CPM is a rare, acute, non-inflammatory, demyelinating disorder characterized by loss of myelin sheath in the neurons of central pons [3]. Extra-pontine structures like the midbrain, thalamus, and cerebellum may also be involved [4]. Demyelination is seen in the structures of the brain that are slow to take the electrolytes that commonly involve the pons or the extra-pontine sites like the midbrain, medulla, and cerebellum [1]. CPM has been linked with extreme variations in serum sodium and plasma osmolarity concentrations [5]. CPM occurs due to the rapid correction of hyponatremia in susceptible individuals like chronic alcoholics, diabetics, and patients suffering from malnutrition [3]. The exact prevalence of the disorder is still not known. The rate of correction associated with CPM is commonly reported as greater than 10 mEq/L per 24 hours or 18 mEq/L per 48 hours [1].

CPM has been linked with increased morbidity and mortality; however, a significant decline has been noted in the last few years due to early detection, timely management and rehabilitation, and more awareness of the disease among internalists and critical care specialists [6]. Despite this, we present a case of a young female who met an unfortunate fate due to medical negligence and error in the rapid correction of hyponatremia leading to locked-in syndrome and CPM.

## Case presentation

A 28-year-old female, married, mother of a four-year-old boy, with no known comorbid presented to the emergency department of a tertiary care hospital with complaints of uncontrolled vomiting, dizziness, and altered sensorium for the last one day. According to the attendant, the patient was in her usual state of health a couple of months back when she developed recurrent urinary tract infections (UTIs) that were managed with oral and systemic antibiotics. Apart from the history of recurrent UTIs, the past medical, surgical, and transfusion history was negative. The patient had a consanguineous marriage and was currently separated from her husband. Her personal history revealed beetle nut addiction since the age of six. The patient had been referred to our institute from another tertiary care setup due to the absence of beds for admission. The patient was managed with the first dose of a broad-spectrum intravenous antibiotic, intravenous hydration, and anti-emetics at the previous hospital.

On admission, the patient was conscious but not alert and unable to follow commands. She had a Glasgow Coma Score (GCS) of eye 4/4 (E4), verbal 1/5 (V1), and motor 4/6 (M6), which was 9/15. On her vitals examination, she was hypotensive with a blood pressure of 90/60 mmHg, tachycardiac at 120 beats/minute, respiratory rate of 18 breaths/minute, and afebrile. On her sub-vitals, she had conjunctival pallor, but the rest of the examination was normal. The neurological examination yielded a decreased GCS, with non-intact higher mental functions. There were no signs of meningeal irritation. The pupillary reflexes were normal and both pupils reacted equally to light. The cardiovascular examination showed tachycardia with normal heart sounds with no added murmurs. The respiratory and abdominal examinations were unremarkable.

The ordered baseline investigations showed anemia, leukocytosis, and mildly deranged renal function tests. The serum sodium level was on the lower limit of normal, and serum potassium was low. The previous hospital's investigations were also reviewed, revealing similar findings except that the serum sodium levels were significantly low. The investigations at baseline and during her course of admission are summarized in Table 1.

**Table 1:** Laboratory investigations during the hospital stay Hb, Haemoglobin; WBC, white blood cell; ALT, alanine aminotransferase; AST, aspartate aminotransferase; ESR, erythrocyte sedimentation rate; CRP, C-reactive protein

Parameter	Prior to admission	Day 1	Day 3	Reference range
Hb	7.6 g/dL	7.6 g/dL	9.2 g/dL	12-16
Hematocrit	30%	31%	34.6%	36-48
WBC count	14.2*10^9^/L	14.5*10^9^/L	8.8*10^9^/L	5-10
Platelets	315*10^9^/L	314*10^9^/L	354*10^9^/L	140-400
Urea	77 mg/dL	76 mg/dL	62 mg/dL	10-50
Creatinine	2.1 mg/dL	2.2 mg/dL	1.8 mg/dL	0.5-1.5
Sodium	111 mEq/L	134 mEq/L	139 mEq/L	136-149
Potassium	3.0 mEq/L	3.1 mEq/L	3.9 mEq/L	3.8-5.2
Chloride	92 mEq/L	92 mEq/L	102 mEq/L	98-107
Total bilirubin	0.34 mg/dL	0.31 mg/dL	0.44 mg/dL	<1
Direct bilirubin	0.12 mg/dL	0.12 mg/dL	0.10 mg/dL	0-0.25
ALT	32 U/L	32 U/L	30 U/L	8-36
AST	35 U/L	34 U/L	37 U/L	0-31
ESR	45 mm/h	44 mm/h	37 mm/h	0-25
CRP	19.7 mg/dL	20 mg/dL	16.7 mg/dL	<5.0
Albumin	3.1 g/dL	2.9 g/dL	2.7 g/dL	3.8-4.4
Urine detailed report	Pus cells 10-15, red blood cells 3-4			

**Figure 1:**
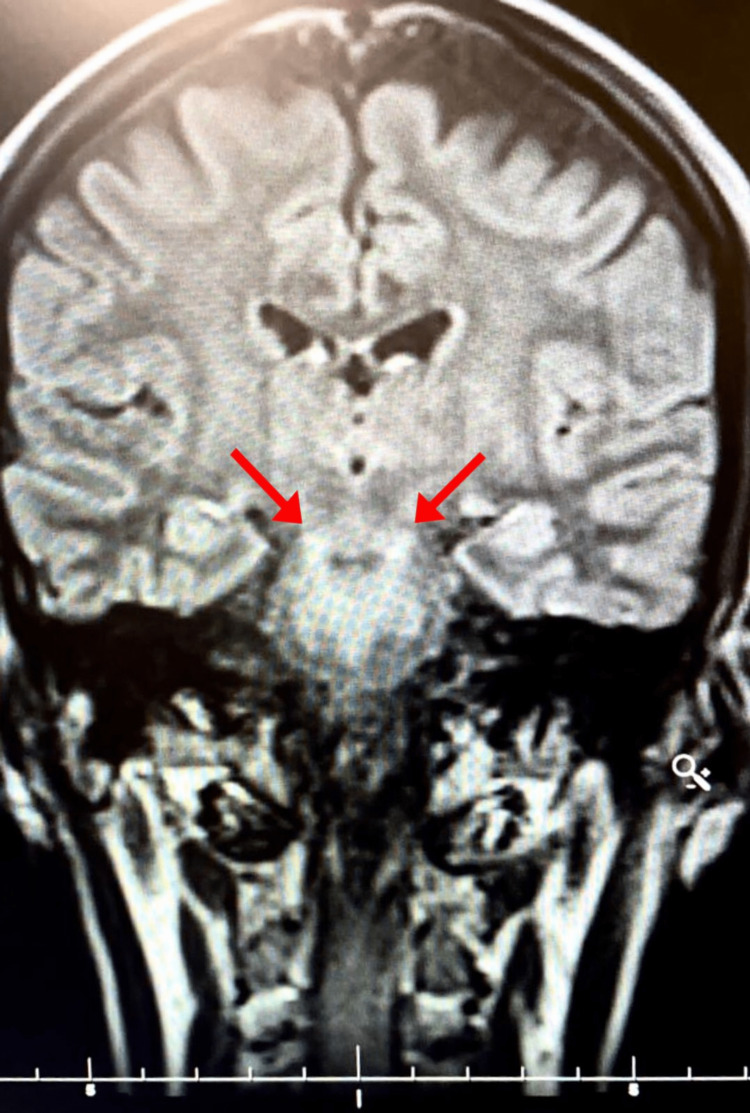
T2-weighted MRI showing pontine hyperintensities (arrows)

A computed tomography (CT) of the brain without contrast was ordered, which was completely unremarkable. The patient started treatment with rehydration, potassium replacement, and intravenous antibiotics. She was also transfused with cross-matched packed red cells to correct her anemia. On the next day, her GCS further deteriorated to E4, V1, M2 (7/15). Her respiratory pattern worsened, and she became breathless having trouble breathing with a respiratory rate of 26 breaths/minute and saturation of 82% on room air. She was immediately oxygenated, and she maintained a respiratory rate of 18 breaths/minute and saturation of 95% at six liters of oxygen. A lumbar puncture was planned to identify the cause of altered consciousness. The results came back to normal. During her stay, the patient’s motor movements completely ceased and the pupils became unreactive, mid-dilated, and fixed. We planned to perform a magnetic resonance imaging (MRI) of the brain with contrast but was delayed until the patient could be weaned off from oxygen. On the fifth admission day, her MRI of the brain was performed after gradual weaning off of oxygen, which yielded hyperintensities in the pons on T2-weighted imaging suggestive of CPM as shown in Figure 1. The records from the previous hospital showed a low sodium level and the baseline sodium concentration at our setup showed sodium at the lower limit of the normal. This was identified as the cause of CPM leading to the characteristic locked-in syndrome.

The patient was slowly weaned off of oxygen requirement. The bad news was broken to the family. She was managed by a multidisciplinary team of neurologists, critical care specialists, psychologists, physiotherapists, and internalists. Counseling regarding supportive management, airway care, physiotherapy, and monitoring of the vitals of the patient was given to the family. The patient’s condition remained static throughout her stay. She was discharged with advice to follow-up. However, on telephonic follow-up, it was discovered that the patient expired 10 days post-admission due to an episode of aspiration pneumonia.

## Discussion

It is commonly seen that CPM has been linked with chronic underlying diseases such as alcoholism, diabetes, malnutrition, malignancy, and human immunodeficiency virus (HIV). Among the causes above, chronic alcoholism is the most common cause [3]. However, in our case, the patient had no underlying chronic illnesses and hyponatremia secondary to intractable vomiting, which was erroneously corrected rapidly, leading to osmotic demyelination in the central pons. Literature review represents that CPM can arise even in cases of normonatremia [7].

The exact pathogenesis of CPM is not known. It is thought to be due to the osmotic changes, and the pons' neurons are highly affected because of their delayed response to the osmotic shift. Due to this, intracellular fluid's rapid movement into extracellular space leads to cell shrinkage and apoptosis [2]. This results in the movement of water out of the neurons in the CSF and the shift of solutes out of the cells [2]. As the pons is the slowest structure of the brain to respond to the osmotic shift, it is the most commonly affected site in CPM.

The patients affected may present with features of unilateral or bilateral facial nerve palsy; quadriplegia; locked-in syndrome; dysarthria; dysphagia; cerebellar features such as ataxia, nystagmus, tremors, confusion, behavioral disturbances; and altered level of consciousness [3]. In a few cases, psychiatric manifestations have also been seen with CPM [3]. Poor prognostic features include low GCS on admission, severe hyponatremia (≤115), low potassium levels, or involvement of the pons [1]. Our patient had features of quadriplegia and facial paralysis presenting as locked-in syndrome. In our patient, rapid rehydration using isotonic saline resulted in the development of CPM, which is a rare cause of CPM. Therefore, it is important to use half-strength saline in the treatment of hypovolemic hyponatremia to prevent rapid correction of serum sodium levels. Another important aspect of the case was the neglect of the laboratory parameters at the time of rehydration to guide therapy. It is imperative to utilize the importance of electrolytes in guiding treatment for dehydration and avoid blind treatment with isotonic saline.

The diagnosis is confirmed by clinical features and radiological imaging. Brain MRI is superior to a CT scan [8]. MRI shows hypointense signals on T1-weighted images and hyperintense signal changes on T2-weighted images in the affected areas [9]. The classical finding is the trident sign and less commonly a piglet sign may be seen [10].

There is no effective cure for the disease. The management is supportive. In the literature review, complete to incomplete reversal of the presenting features have also been documented. In a report by Feng XM et al., there was a complete reversal of the clinical features at one-month follow-up [3]. A few cases also represent the use of intravenous immunoglobulins and plasmapheresis for the reversal of symptoms with positive results [1]. Other measures of supportive care include maintenance of the airway and prevention of complications like deep vein thrombosis and aspiration pneumonia, one of which led to the fatal demise in our case [4]. Our patient met an unfortunate fate due to the poor prognostic features present upon presentation that included serum sodium level <115 (111 mEq/L), low serum potassium level on admission, low GCS, and involvement of the pons on MRI imaging. The focus should be targeted on the prevention of CPM. As the guidelines suggest, sodium correction should be around 10 mmol/L/day for both acute and chronic hyponatremias [11].

## Conclusions

CPM is a rare demyelinating disorder with a poor prognosis and increased mortality. It is a preventable disease that requires creating awareness regarding the syndrome and utilizing the correct guidelines on the correction of hyponatremia, which is the common precipitating agent. Our case is of significance because, in the absence of common precipitating factors, our patient developed classical clinical and radiological features of CPM due to the failure of correct interpretation of the lab reports and blind treatment of vomiting with rapid intravenous rehydration with isotonic saline.
